# γ-Synuclein Antibodies Have Neuroprotective Potential on Neuroretinal Cells via Proteins of the Mitochondrial Apoptosis Pathway

**DOI:** 10.1371/journal.pone.0090737

**Published:** 2014-03-03

**Authors:** Corina Wilding, Katharina Bell, Sabine Beck, Sebastian Funke, Norbert Pfeiffer, Franz H. Grus

**Affiliations:** Experimental Ophthalmology, Department of Ophthalmology, Medical Center of the Johannes Gutenberg University Mainz, Mainz, Germany; Massachusetts Eye & Ear Infirmary, Harvard Medical School, United States of America

## Abstract

The family of synuclein proteins (α, β and γ) are related to neurodegenerative disease e.g. Parkinson disease and Morbus Alzheimer. Additionally, a connection between γ-synuclein and glaucoma, a neurodegenerative disease characterized by a progressive loss of retinal ganglion cells, which finally leads to blindness, exists. The reason for the development of glaucoma is still unknown. Recent studies evaluating the participation of immunological components, demonstrate complex changed antibody reactivities in glaucoma patients in comparison to healthy people, showing not only up-regulations (e.g. alpha-fodrin antibody) but also down-regulations (e.g. γ-synuclein antibody) of antibodies in glaucoma patients. Up-regulated antibodies could be auto-aggressive, but the role of down-regulated antibodies is still unclear. Previous studies show a significant influence of the serum and the antibodies of glaucoma patients on protein expression profiles of neuroretinal cells. The aim of this study was to investigate the effect of γ-synuclein antibody on the viability and reactive oxygen species levels of a neuroretinal cell line (RGC-5) as well as their interaction with cellular proteins. We found a protective effect of γ-synuclein antibody resulting in an increased viability (up to 15%) and decreased reactive oxygen species levels (up to −12%) of glutamate and oxidative stressed RGC-5. These can be traced back to anti-apoptotic altered protein expressions in the mitochondrial apoptosis pathway indicated by mass spectrometry and validated by microarray analysis such as active caspase 3, bcl-2 associated-x-protein, S100A4, voltage-dependent anion channel, extracellular-signal-regulated-kinase (down-regulated) and baculoviral IAP repeat-containing protein 6, phosphorylated extracellular-signal-regulated-kinase (up-regulated). These changed protein expression are triggered by the γ-synuclein antibody internalization of RGC-5 we could see in immunohistochemical stainings. These findings let us assume a novel physiological function of γ-synuclein antibodies and give insights in the role of autoantibodies in glaucoma. We hypothesize that the down-regulation of autoantibodies found in glaucoma patients lead to a loss of protective autoimmunity.

## Introduction

Synucleins are a family of small, cytosolic proteins consisting of α-, β- and γ-synuclein. They are abundant in neuronal tissues [1] and associated with the pathogenesis of neurodegenerative diseases. Although physiological functions of synucleins are not entirely understood, there are hints that α, β and γ-synuclein possess chaperon like activity [2]. Studies show a mutated form of α-synuclein in patients with autosomal dominant Parkinson disease [3] and as a component of plaques in Alzheimer patients [4], [5]. Furthermore α-synuclein is a component of Lewy bodies in Parkinsons disease [6]. All synucleins are expressed in retina and optic nerve [7]. γ-synuclein is involved in neurodegenerative and ocular diseases [8], [9] and is highly expressed in retinal ganglion cells (rgc) [10]. In comparison to healthy people, the optic nerve head and retina of glaucoma patients show different γ-synuclein localizations [9], [11]. Glaucoma is a heterogeneous neurodegenerative disease defined by a progressive loss of rgc, optic nerve degeneration and progressive visual field loss, which finally can lead to blindness [12]. Although glaucoma is one of the most common causes for blindness worldwide [13] the pathogenesis is still unknown. A major risk factor is an elevated intraocular pressure, but 30% of patients don't show this manifestation [14]. Studies suggest an immunological component in the pathology of glaucoma. An increased occurrence of paraproteins and autoantibodies against nuclear antigens like Sjögren's syndrom A, was demonstrated in glaucoma patients [15]. Furthermore, studies show not only up-regulated, but also down-regulated antibodies (abs) in glaucoma patients. In the serum and aqueous humor of glaucoma patients general complex autoantibody patterns against retinal and optic nerve antigens were found [16], [17] but also more specific autoantibody changes such as an up- regulation of abs against e.g. alpha foldrin [16], [17] and Hsp 70 [18], and a down-regulation of abs against αB Crystallin and Vimentin [18] leading to the conclusion that there is a role for the autoantibodies in the pathogenesis of glaucoma. Previous studies incubating neuroretinal cells with the serum and the abs of glaucoma patients found changed protein expression patterns in cells incubated with glaucoma serum in comparison to serum from healthy people [19]. Furthermore the cells reacted differently towards the serum after removal of IgG abs [19]. These results underline the hypothesis that changes in the autoantibodies could play a role in the pathogenesis of the disease.

One autoantibody down-regulated in glaucoma patients is targeted against γ-synuclein. This study aimed to investigate, which effect the down-regulated ab against γ-synuclein has on stressed neuroretinal cells.

## Materials and Methods

### Chemicals

Dulbeco's modified eagle medium (DMEM), fetal calf serum (FCS), penicillin, streptomycin, glutamate, phosphate buffered saline (PBS), crystal violet, 2′,7′-dichlorodihydrofluorescein-diacetate (DCFH-DA), 0,25% Triton-X-100, bovine serum albumin (BSA), cell dissociation solution (CDS), dodecyl-D-β- Maltosid, ammoniumbicarbonat (AB) were purchased from Sigma Aldrich (St. Louis, MO). L-alanyl-L-glutamin was purchased from Biochrom AG (Berlin, Germany). H_2_O_2_ and paraformaldehyde was obtained from Carl Roth GmbH (Karlsruhe, Germany), staurosporine was purchased from Calbiochem (San Diego, CA). Ethanol, acetonitril (ACN), trifluoroacetic acid (TFA) and formic acid were purchased from Merck (Darmstadt, Germany), wheat germ agglutinin from Invitrogen (Carlsbad, U.S.A.) and BCA Pierce Protein Assay kit and Dylight 649 was purchased from Fisher scientific (Waltham, MA.). Trypsin was from Promega (Mannheim, Germany), HPLC H_2_O from Applichem (Darmstadt, Germany) and C-18 ZipTips was purchased from Millipore (Billerica, MA). The used abs were listed in table 1.

**Table 1:** **List of used abs.**

Antibody	Species	UniProt accession	Distributor
Polyclonal anti active caspase 3	Rabbit	P42574	Antibodies-online
Polyclonal anti PRA1 family protein 2 (JM4)	Rabbit	O60831	Antibodies-online
Polyclonal anti Bcl2-assiciated x protein (BAX)	Rabbit	Q07812	Antibodies-online
Monoclonal anti Bcl-2 antagonist of cell death (BAD)	Mouse	Q92934	Abcam
polyclonal anti phosphorylated extracellular regulated protein 1 ab (p-ERK1)	Goat	Not available	Santa Cruz Biotechnology
monoclonal anti extracellular regulated protein 1 (ERK1)	Recombinant	P27361	Abcam
Monoclonal anti voltage-dependent anion channel (VDAC)	Mouse	P21796	Abcam
Polyclonal anti baculoviral IAP repeat containing 6 (BIRC6)	Rabbit	Q9NR09	Abcam
polyclonal anti Caspase 9	Rabbit	Not available	Bioworld Technology
Monoclonal anti S100A4	Mouse	P26447	Abcam
polyclonal anti myoglobin ab	Rabbit	P02144	Abcam
polyclonal anti γ-synuclein ab	Sheep	O76070	Abcam
polyclonal anti γ-synuclein ab	Goat	Not available	Santa Cruz Biotechnology
anti sheep IgG-H&L conjugated with FITC	rabbit	Not available	Abcam

### Cell culture

RGC-5 cells were provided by Dr. Neeraj Agarwal and are transformed with a ψ2E1A virus [20]. RGC-5 are of mouse origin, representing a neuronal precursor cell line [21]. The cells were grown in 75 cm^2^ culture flasks in DMEM supplemented with 10% FCS, 100 U/ml penicillin, 100 U/ml streptomycin and 4% L-alanyl-L-glutamin and cultivated in a humidified incubator at 37°C and 5% CO_2_. They were passaged when 80% confluent.

### Cell treatment with γ-synuclein abs and different stress factors

RGC-5 cells were seeded in 24 well plates at a density of either 45000 or 40000 cells per well (24 h or 48 h experimental duration) and grown over night. The cells then were preincubated with different concentrations of goat polyclonal anti-γ-synuclein abs (0.005, 0.1, 0.5, 1, 5 µg/ml) and subsequently incubated with different stress factors. Oxidative stress was induced by incubating the cells with 50 µM H_2_O_2_ for 1 h. Staurosporine was applied at a concentration of 1.5 µM for 5 h and 20 mM glutamate for 24 h to introduce apoptotic stress. In order to detect the specificity of the results the experiments were also performed with different concentrations of rabbit polyclonal myoglobin abs and either stressed with 1.5 µM staurosproine, 20 mM glutamate or 50 µM H_2_O_2_ (n for all experiments = 4).

### Cell viability test

Cell viability was assessed with crystal violet staining. After fixing the cells with 3% paraformaldehyde for 15 min and rinsing with PBS, the cells were stained with 0.1% crystal violet solution for 20 min. Excess stain was washed three times with distilled water. After the plates were dried, the bound stain was resolved with 70% ethanol for 2 h and the supernatants were read in a Multiscan ascent plate reader (Thermo scientific, Waltham, MA) with a 570 nm filter. The absorption was expressed as a percentage of the control cells only treated with the stress factors. An unpaired student's t-test was used to compare the data obtained and was calculated with Statistica (StatSoft, U.S.A.). A p-value <0.05 is described as significant and a p-value <0.01 as highly significant.

### ROS-test

To quantify ROS we used DCFH-DA. Through intracellular esterase and ROS the non-fluorescence stain DCFH is converted to the fluorescent stain DCF. Cells were loaded with 10 mM DCFH-DA in a humidified incubator of 37°C, 95% air and 5% CO_2_ for 15 min. After replacing the medium, 50 µM H_2_O_2_ was added for 1 h in order to generate ROS. The fluorescence was measured by using the microplate reader fluoroscan ascent (Thermo scientific) with excitation/emission wavelengths of 485/538 nm. The fluorescence was expressed as a percentage of the control cells treated only with 50 µM H_2_O_2_. The ROS-level was normalized by measuring the viability of the cells in the same well. An unpaired student's t-test calculated with Statistica was used to compare the data obtained. A p-value <0.05 is described as significant and a p-value <0.01 as highly significant.

### Immunocytochemical staining

RGC-5 cells were grown in μ-slide IV (Ibidi GmbH, München, Germany) over night and subsequently washed with PBS. Then the cells were fixed with 3% paraformaldehyde (15 min), incubated with 0.25% Triton-X-100 in PBS (12 min), washed 3× with PBS and treated with 1% BSA (20 min). After incubating the cells with 2 µg/ml sheep polyclonal anti-γ-synuclein abs over-night they were gently washed 3× with PBS and incubated with rabbit anti sheep IgG-H&L conjugated with FITC for 1.5 h. They were visualized with Leica fluorescence microscope using Lucia G/F software after washing them with PBS. To investigate the ab uptake in living cells the cells were incubated with 15 µg/ml sheep polyclonal anti-γ-synuclein abs and washed with PBS. The cell membrane was visualized using wheat germ agglutinin.

### Mass spectrometry analysis

#### Cell lysate preparation

For proteomic analysis cells were grown in 60×15 mm cell culture dishes and incubated with 0.5 µg/ml goat polyclonal anti-γ-synuclein abs. Control cells were incubated without abs. The cells were washed with PBS, detached from the cell culture dish with CDS and lysed by freezing at −80°C, adding 0.1% Dodecyl-D-β- Maltosid and treatment with an ultrasonic bath for 1 min. After centrifugation, the supernatant was used to determine the protein concentration by BCA Pierce Protein Assay kit.

### SDS PAGE separation and In-gel digestion

To separate the proteins a denaturing gel electrophoresis was performed. Each lane was cut into 17 pieces, incubated with ACN and AB and dried in a concentrator. Following this, the pieces were tryptically digested (0.7 µg Trypsin in 80% HPLC H_2_O, 10% ACN, 10% AB) over night. The supernatant was collected and the remaining proteins were dissolved with an extraction buffer (38% HPLC H_2_O, 0.2% formic acid, 60% ACN) for 30 min. Both supernatants were pooled, dried in a concentrator and acidified with 0.1% TFA. C-18 ZipTips (Millipore, Billerica, MA) were used to clean the samples according to a protocol from the manufacturer. The samples were then dried and frozen at −20°C until further analysis.

### LC-ESI/MS for protein identification

Analysis of peptides was performed with a capillary LC-ESI-MS system consisting of a BioBasic C-18 precolumn (30 mm×0.5 mm, Thermo Scientific) and a BioBasic C18 analytical column (150 mm×0.5 mm, Thermo Scientific).The whole system was additionally protected by an A 316 0.5 µm online precolumn filter (Upchurch Scientific, Washington, U.S.A.). As solvent delivery system a Rheos Allegro HPLC Pump (Thermo Scientific) was used. The pump flow rate was adjusted to 200 µl/min, which was reduced to a column flow of 10 µl/min by use of an M-472 graduated micro-split valve (Upchurch, Scientific) (Running buffer A: 98%H_2_O, 1.94% ACN, 0.06% methanol, 0.05% TFA and running buffer B: 95% ACN, 3%methanol, 2% H_2_O and 0.05% TFA). A linear gradient of 80 min was performed (0–47 min: 0–100%B, 47–49 min: 100% B, 49–58: 100%–0% B, 58–80 min: 0%B). Equilibration gradients of 30 min were run between the samples by injecting 80% ACN to the system to prevent sample-to-sample carry over. Mass spectra were obtained using an LTQ OrbitrapXL. The full-scan mass spectra (from m/z 300–2000) were acquired with a resolution of 30.000. For MS/MS analyses ions were isolated with an isolation width of 1 m/z and for fragmentation a collisions induced dissociation was performed in the iontrap with a normalized collision energy of 35, an activation of 0.25 (m/z) and an activation time of 30 ms. A dynamic exclusion was also applied to minimize acquisition of redundant MS/MS using following condition: repeat duration 30 s and exclusion duration 90 s. Mass spectra were recorded in the “profile” mode to allow quantification in MaxQuant (Max Planck Institute of Biochemistry, Martinsried, Germany).

### Data processing

The obtained mass spectra were used for an identification and quantification with Maxquant. As fixed modification we set carbamidomethylation. The tolerance in mass precision for MS/MS was 20 ppm and 0.5 Da. The protein and peptide false discovery rate were set at 0.01, the minimum peptide length was 6 amino acids and the minimum unique peptides were set at 2. The evaluation was implemented with Ingenuity Pathway Analysis (IPA) software to investigate biological networks and pathways. In the pathway analysis we included only proteins with a 2-fold changed expression in γ-synuclein ab treated cells. The statistical significance of each pathways were calculated by IPA using a Fisher Exact test (p<0.05).

### Protein microarray

A set of specifically chosen abs (Table 1) against proteins of the mitochondrial apoptosis pathway were used to create an ab microarray in our laboratory. The abs were diluted in PBS and spotted on a nitrocellulose slide using an array spotter (Scienion, Germany). Each ab spot was replicated 3 times. Cells were preincuabted with 0.5 µg/ml goat polyclonal anti γ-synuclein abs for 3 h and subsequently lyzed and protein concentrations determination was performed as described above. Control cells were incubated without abs. The cell lysates (n = 12) were then labeled with Dylight 649 for 1 h in the dark and quenched with Tris-HCl for 1 h. The microarray slides were blocked with 5% BSA in 0.5% Tween-PBS for 1 h, washed 3× with 0.5% Tween-PBS and subsequently were incubated with the labelled cell lysates for 2.5 h. After washing the slides 3×, the arrays were digitalized with our array scanner (Aviso GmbH, Germany). For data analysis spot intensity was quantified with ImaGene 5.0 Software (BioDiscovery, Waltham, MA). Defect spots were manually flagged and the signal median of 3 replicate spots were averaged. The statistics were calculated with Statistica using an unpaired students t-test (p<0.05). All procedures were performed in our laboratory.

## Results

### Effect of γ-synuclein abs on stressed RGC-5

**Figure 1:**
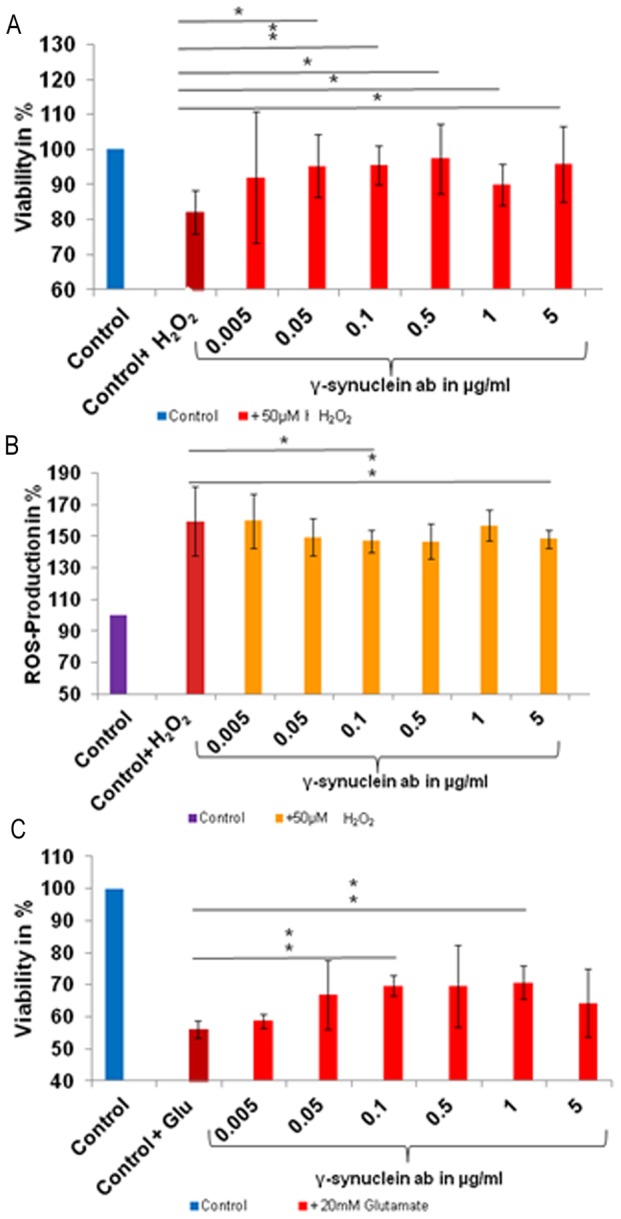
Viability and ROS-level of γ-synuclein ab treated and stressed RGC-5. RGC-5 were preincubated with different γ-synuclein ab concentrations and additionally stressed with 20 mM glutamate for 24 h and 50 µM H_2_O_2_ for 1 h. Cell viability as well as ROS-level were determined using crystal violet and DCFH-DA (* = p<0.05; **p<0.01) **A**: Increased significant and high significant cell viability were measured after the cells were preincubated with 0.05–5 µg/ml γ-synuclein abs and additionally stressed with H_2_O_2_. **B**: Significant and high significant decreased ROS production was measured, when the cells were preincubated with 0.1 and 5 µg/ml γ-synuclein abs. **C**: Increased cell viability in a range of 0.05–5 µg/ml γ-synuclein abs were obtained after the cells were stressed with glutamate, whereby the results of 0.1 and 1 µg/ml are high significant.

The effect of γ-synuclein abs was determined by viability and reactive oxygen species (ROS)-tests. Dose response studies identified the ideal concentration of 50 µM H_2_O_2_ for 1 h, 1.5 µM staurosporine for 5 h and 20 mM glutamate for 24 h (data not shown). These concentrations and incubation times were used in all experiments. We detected a significantly increased cell viability of up to 15% (p<0.05) when preincubating the cells with 0.05 (p = 0.026), 0.5 (p = 0.022), 1 (p = 0.036) and 5 µg/ml (p = 0.045) γ-synuclein abs and additional stressing with H_2_O_2_ in comparison to the control cells only treated with H_2_O_2_ (Figure 1A). We found highly significant increase of cell viability of 13% when preincubating the cells with 0.1 µg/ml γ-synuclein abs (p = 0.0032). The same concentrations of 0.1 and 5 µg/ml γ-synuclein abs also showed a significant and highly significant decrease of ROS-level of −12% (p = 0.010) and −11% (p = 0.008) in H_2_O_2_ stressed RGC-5 (Figure 1B). No significant effect was found when the cells were incubated with lower concentrations of the abs.

Staurosporine was used to induce apoptosis in RGC-5. No changes in the cell viability were found when preincubating the cells with different concentrations of γ-synuclein abs and additional stress with staurosporine (data not shown).

We detected an increased viability of cells incubated with 0.1 (p = 0.0002) and 1 µg/ml (0.001) γ-synuclein abs and stressed with glutamate of up to 14% in comparison to control cells which were only treated with glutamate (Figure 1C).

To validate the specific protective effect of γ-synuclein abs the same experiment was performed with anti myoglobin abs. Myoglobin is a specific heart muscle protein which is responsible for the intramuscular oxygen transport. We could not detect any significantly changed viability when RGC-5 were preincubated with different concentrations of myoglobin abs and additionally stressed either with staurosporine, glutamate or H_2_O_2_ in comparison to untreated cells (data not shown).

### Expression of γ-synuclein and γ-synuclein ab uptake in RGC-5

**Figure 2:**
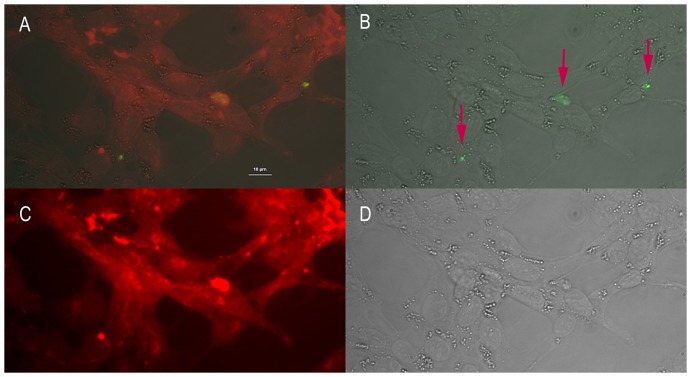
γ-synuclein ab uptake of RGC-5 revealed by indirect immunfluorescence. Living cells were preincubated 3γ-synuclein abs and then fixed, permeabilized, blocked and stained with rabbit anti sheep IgG-H&L conjugated with FITC. Cell membrane was stained with wheat germ agglutinin conjugated with Rhodamin. **A**: Bright light merged with corresponding fluorescence and cell membrane staining microscopy. **B**: Corresponding fluorescence micrograph merged with bright light. **C**: Cell membrane microscopy. **D**: Bright light microscopy

To determine, whether RGC-5 cells express γ-synuclein and whether living cells bind anti γ-synuclein abs an indirect immunofluorescence staining was performed. The permeabilized cells showed, as also presented in former studies analyzing γ-synuclein expression in retinal ganglion cells, a cytoplasmatic staining of γ-synuclein (Figure S1). Furthermore we detected γ-synuclein ab uptake into living cells (Figure 2).

### Mass spectrometry analysis

**Figure 3:**
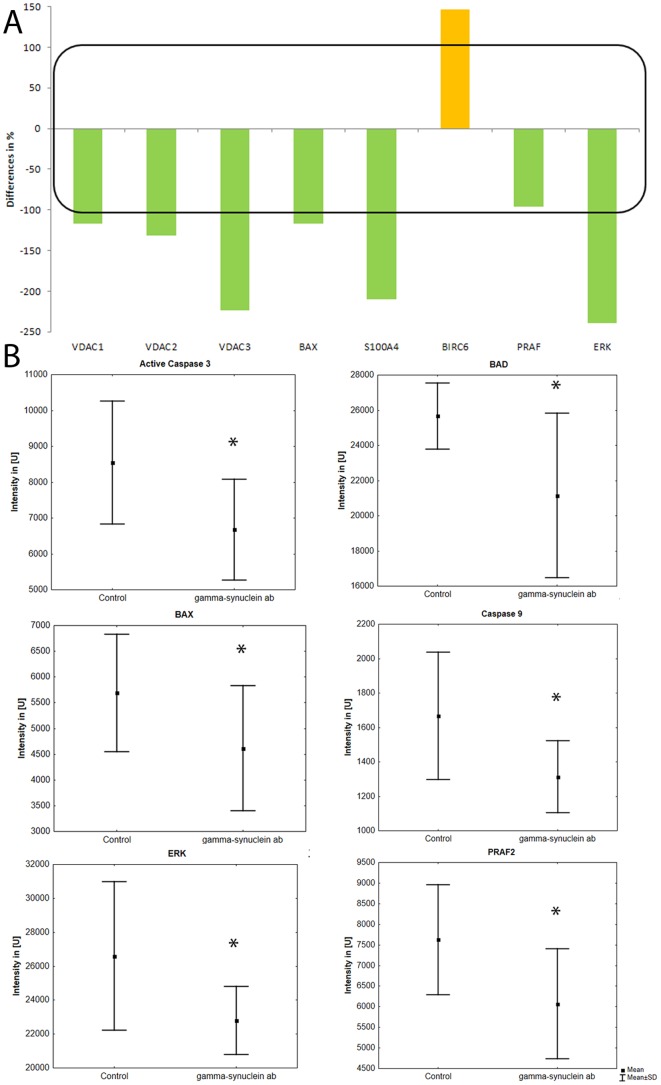
Regulation of mitochondrial apoptosis associated proteins. RGC-5 cells were preincubated with 0.5 µg/ml γ-synuclein abs and subsequently lyzed, tryptically digested before protein analysis was performed. **A**: Regulation of mitochondrial apoptosis associated proteins. Protein analysis was performed via capillary LC-ESI-MS system. Quantification of the proteins was realized with MaxQuant. The differences were calculated in comparison to control cells, which were untreated. **B**: Regulation of mitochondrial apoptosis associated proteins. Protein analyses were performed via Microarray. The differences were calculated in comparison to control cells, which were untreated. (n = 12, * = p<0.05; **p<0.01)

Using mass spectrometry analyses, the effect of γ-synuclein abs on the proteins of the cells and the determination of possibly involved pathways were investigated in cells incubated with γ-synuclein abs in comparison to untreated cells. 1110 proteins were identified of which 200 were significantly differently expressed (>2 fold up- or <2 fold down-regulated) in the ab-treated cells (Table S1). These proteins were analyzed with IPA and classified in 34 significant canonical pathways. Among these pathways was the intrinsic apoptotic pathway, showing 6 significantly differently expressed proteins such as BAX, BIRC6, S100A4, VDAC 1/2/3, ERK1/2, which are involved in the regulation of the mitochondrial apoptosis pathways and were regulated in an anti-apoptotic manner. BAX, VDAC 1/2/3 and S100A4 were significant down-regulated and BIRC6 were significant up-regulated in cells treated with γ-synuclein abs (Figure 3A).

### Microarray analyses

To validate the results of the mass spectrometry analysis, microarray analyses were performed. The analysis showed a confirmation of the mass spectrometric results. BAX (p = 0.035), PRAF2 (p = 0.009) and ERK1/2 (p = 0.018) were significantly and highly significantly down-regulated in γ-synuclein ab treated RGC-5. The tendency of VDAC and S100A4 correlates with the results of the mass spectrometric analysis (Figure S2). Other, additionally analysed proteins of the mitochondrial apoptosis pathways were significantly down-regulated e.g. active caspase-3 (p = 0.008), caspase-9 (p = 0.001) and BAD (p = 0.014) (Figure 3B).

## Discussion

### Protective effect of γ-synuclein abs on stressed RGC-5 cells

This study demonstrates a protective effect of different γ-synuclein ab concentrations on glutamate and H_2_O_2_ stressed neuroretinal cells, which result in increased viability and decreased ROS-levels. The lowest concentration of γ-synculein abs shows no effect on the viability of the cells. We were able to detect a protective effect in cells preincubated with γ-synuclein ab in the range from 0.005 to 5 µg/ml. Not all concentrations show a significant effect, however tendencies which suggest a protective effect are distinguishable. A dose response effect as well as a negative effect of high γ-synuclein ab concentrations couldn't be observed. Studies show that ab-uptake into cells can be saturable [22]. Our immunohistochemical staining results showing small amounts of abs in the cells at one defined time point support the assumption that ab uptake of the used cells is restricted. Furthermore, high ab concentrations not necessarily have a negative effect, as other studies could show that even high concentrations of ab, also internalized by cells, do not have a negative influence on the viability of cells [23]. This could be due to the fact that the binding partners of the abs are saturated and further abs cannot be bound and therefore have no additional effect. When using unspecific abs such as anti-myoglobin abs no protective or negative effect was detected. Studies demonstrate an impact of γ-synuclein on apoptotic pathways in RGC. Knocking down γ-synuclein in RGC-5 leads to decreased viability through the regulation of kinases and phosphatases [11]. In general, the effect of changes in γ-synuclein expression either in vivo or in vitro shows opposing results. In vivo studies show that an up-regulation of γ-synuclein can lead to neurodegeneration [24], which stands in contrast to other reports demonstrating that an overexpression of γ-synuclein has no negative effect [25] whereas other studies show that there is no effect on neuronal cells when inactivating γ-synuclein [26]. Additionally studies show that γ-synuclein can participate in signal transduction pathways. In Y79 cells over-expression of synoretin, the bovine orthologous of γ-synuclein, induces increased MAPK activity as well as its downstream effector Elk-1. MAPK are involved in the transmission of extracellular signals to intracellular targets and affect many cellular processes, e.g. cell survival, cell proliferation, gene expression and apoptosis [27]. These results demonstrate that γ-synuclein can influence cell viability, signal transduction pathways and also stress response. Therefore we hypothesize that the binding of γ-synuclein ab on its antigen γ-synuclein can alter the functions of the protein, which, when applied in low doses, results in a protective effect against H_2_O_2_ and glutamate.

### γ-synuclein ab uptake in RGC-5

In order to evaluate the mechanism of the protective effect in more detail, immunohistochemical staining was performed. The staining confirmed former studies which show a binding of the ab in the cytoplasma of permeabilised RGC-5 (Figure S1) [10]. An uptake of γ-synuclein abs in vesicles of living cells could also be observed (Figure 2). Several studies in vivo as well as in vitro have been able to demonstrate ab uptake into cells, e.g. neuronal cells [28] [29] [30]. Uptake mainly uses the process of endocytosis, which can occur very quickly and at different time points. We couldn't detect an accumulation or the uptake of a huge amount of γ-synuclein ab, which could be caused by a restricted ab uptake, also demonstrated for other cells [22]. Another possibility could be the intracellular degradation of the ab, e.g. via transportation to lysosomes or to the Golgi Apparatus [31]. Degraded abs then cannot be detected using a secondary ab against IgG. Furthermore, studies also are able to show ab recycling and transportation to the extracellular space [32]. Abs are large proteins with a molecular weight of 140–150 kDa. The mechanisms by which abs can be transported into cells or translocated into the nucleus or other organelles are not understood very well. Next to endocytosis, different hypothesis exist on how abs can penetrate into living cells. Ab penetration mediated through the Fc receptor was described [33] and also the uptake of anti-DNA abs into living cells, mediated by myosin1 [34]. The internalized anti-DNA abs interact with DNAse1 within the cytoplasm and inhibit its enzymatic activity. Furthermore the transfer of anti-DNA abs into the nucleus and their return transport to the cell surface was demonstrated [34] and ab uptake by clathrin-associated-vesicles, a specific type of endocytosis, has been described [35].

### Influence of γ-synuclein abs on mitochondrial apoptosis pathways

**Figure 4:**
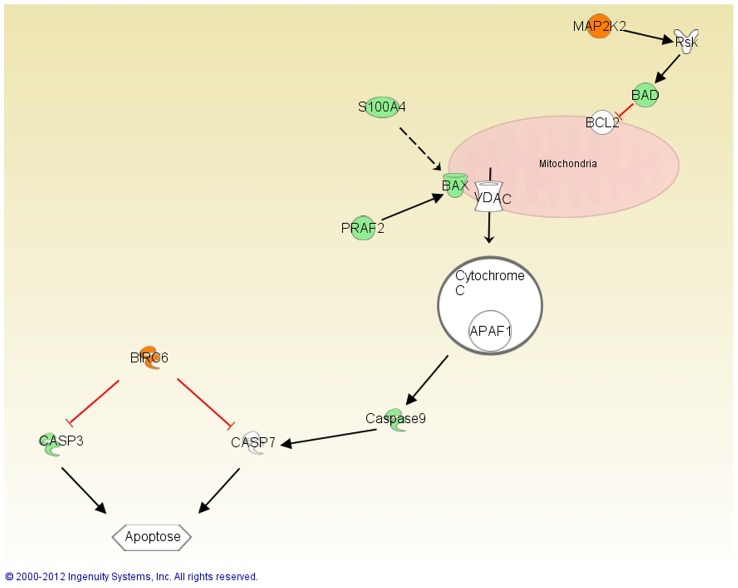
Overview of changed proteins of the mitochondrial apoptosis pathway. This pathway was performed with IPA. The red colored proteins demonstrate that the protein is up-regulated and the green colored that they are down-regulated. Red arrows represent a direct inhibitory function and the black arrows direct activation function. The dotted arrow represents indirect interaction via transcriptional regulation.

The mass spectrometric as well as the microarray analysis demonstrate changed protein expressions of mitochondrial apoptosis pathway proteins in γ-synuclein ab treated RGC-5 such as BAX, BIRC6, S100A4, BAD, PRAF2, active Caspase-3, Caspase-9 and VDAC 1/2/3 (Figure 4). All these proteins are regulated in an anti-apoptotic manner and therefore most likely participate in the protection of RGC-5 against glutamate and H_2_O_2_.

Pro-apoptotic BAX belongs to the Bcl-2 family and plays an important role in the intrinsic apoptotic pathway through binding mitochondrial VDAC, which leads to the release of cytochrome c and finally to the initiating of apoptosis [36]. In an elevated intraocular pressure mouse glaucoma model the expression of BAX was increased in hypertensive eyes in comparisons to control eyes [37]. Also, a BAX deficiency in DBA/2J mouse protects RGC from cell death [38]. The expression of BAX is regulated by transcription factor p53 which in turn is regulated by S100A4, down-regulated in γ-synuclein ab treated cells. S100A4 induction in a murine non-metastatic adenocarcinoma cell line leads to an increased expression of BAX and thereby to increased apoptosis [39].

The anti-apoptotic protein BIRC6 belongs to the inhibitor of apoptosis (IAP) family and is up-regulated in γ-synuclein ab treated RGC-5 (Figure 4A). BIRC6 is up-regulated in tumors and can inhibit active caspase-3 [40]. Studies show that over-expression of BIRC6 in mammalian cells inhibits apoptosis [41]. In an ocular hypertensive glaucoma model the over-expression of BIRC4, another member of the IAP family, promotes optic nerve axon survival [42]. VDAC 1/2/3, significantly down-regulated in this study, play an important role in apoptosis-initiation and are located on the outer mitochondrial membrane. They participate in energy balance regulation as well as in the release of pro-apoptotic factors. Studies show that a reduction of VDAC1 levels in endothelial cells attenuates endostatin induced apoptosis [43]. Other proteins, such as active caspase-3, caspase-9 and BAD were down-regulated in this study whereas the active form of ERK called p-ERK1/2 was up-regulated in γ-synuclein ab treated RGC-5. The well characterized ERK pathway transfers signals from different membrane receptors into the nucleus. It is composed of different kinases which activate ERK1. Activated ERK1, which is increased in RGC-5 treated with γ-synuclein abs, is able to phosphorylate many cytoplasmic as well as nuclear targets, which leads to cell proliferation [44], [45]. An experimental rat glaucoma model shows that the activation of ERK leads to increased survival of rgc after ocular hypertension surgery [46]. A MEK-ERK survival pathway is described, whereby activated MAPK participate in the phosphorylation of BAD and promote cell survival (Figure 4) [47]. BAD is a pro apoptotic member of the Bcl-2 family and participates in the initiation of apoptosis. Studies assume an involvement of BAD and active caspase-3 in glaucoma [48], [49], which leads to the cellular protein cleavage and apoptosis [50].

Studies show that γ-synuclein is able to bind transcriptional factors and modulate the transcription of genes and factors such as JunB, MECP2, CREB1 and ATF3 [51], [52]. Furthermore γ-synuclein can interfere with the mitochondrial apoptosis pathway through transcriptional regulation of kinases and phosphates, which control the phosphorylation status of BAD [11]. Other studies analyzing ab functions, such as hsp27 ab, show that the binding of hsp27 abs on its antigen leads to a modulation of hsp27 which ends in an inactivation or inhibition of the protective function [53]. Anti- recoverin abs were also detected to be internalized in photoreceptor cells and bipolar cells of the retina and trigger apoptotic cell death [54]. Therefore it is imaginable that internalized γ-synuclein abs bind their antigen and alter its function. The modulated function of γ-synuclein could lead to a changed binding of transcription factors and therefore to a changed expression of mitochondrial apoptosis proteins. Future experiments are needed to provide more information about the exact mechanisms.

### Correlation with findings of clinical studies

Beside other altered ab reactions, clinical studies show a lower concentration of γ-synuclein ab in the serum of glaucoma patients. Many up-regulated abs found in classical autoimmune disease have auto-aggressive potential, for example in Myasthenia gravis where the binding of abs against nicotine acetylcholine receptor leads to muscular weakness [55], [56]. In contrary we hypothesize that the down-regulation of autoantibodies in glaucoma patients could reflect a loss of protective autoimmunity. Studies found autoantibodies in the serum of healthy people which have protective effects [57]. In the serum of patients suffering from Alzheimer's disease reduced autoantibodies against Aβ can be detected [58], which have a protective effect by inhibiting oligomerization of Aβ peptides in an animal model [59]. Furthermore autoantibodies against α- synuclein were found in patients with inherited Parkinson's disease which possibly also are part of a protective reaction [60].

## Conclusion

We hypothesize that the dysbalance of the natural autoantibodies can alter the regulatory functions and therefore can make cells, e.g. rgc more vulnerable to external stress factors such as an elevated pressure.

In summary we can show protective effects of ab against γ-synuclein on neuroretinal cells. These protective effects are most possibly mediated via changes in the mitochondrial apoptosis pathway, which are triggered by the uptake of the ab into the cell. Not only in glaucoma, but also in Alzheimers disease, down-regulated autoantibodies were detected, which seem to lead to a loss of protective effects. Therefore and due to the fact that autoantibodies not only have destructive but also regulatory effects we assume that autoantibodies down-regulated in glaucoma patients lead to a reduction of regulatory functions and therefore to a loss of protective regulation. The sum of changes of the abs could therefore, in a long term, lead to an increased vulnerability of retinal ganglion cells for external stress factors, e.g. an elevated intraocular pressure.

## Supporting Information

Figure S1 **Expression of γ-synuclein in RGC5 revealed by indirect immunofluorescence** RGC-5 cells were fixed, permeabilised, blocked and incubated with sheep polyclonal anti γ-synuclein abs. Subsequently the cells were incubated with rabbit anti sheep IgG-H&L conjugated with FITC. Nuclei staining were performed with DAPI and cells were visualized with a fluorescence microscope. γ-synuclein was expressed in all cells and it seems to be distributed in the cytoplasm.
(TIF)

Figure S2 **Regulation of mitochondrial apoptosis associated proteins.** RGC-5 cells were preincubated with 0.5 µg/ml γ-synuclein abs and subsequently lyzed, tryptically digested before protein analysis via Microarray was performed. The differences were calculated in comparison to control cells, which were untreated. (n = 12, * = p<0.05; **p<0.01).
(TIF)

Table S1 **Significant protein changes in γ-synuclein antibody treated RGC-5.**
(DOC)
