# Supplementary figures and images for: γ-Synuclein Antibodies Have Neuroprotective Potential on Neuroretinal Cells via Proteins of the Mitochondrial Apoptosis Pathway
Source: PLoS One. 2014 Mar 3;9(3):e90737. doi: 10.1371/journal.pone.0090737 (PMC3940944; doi:10.1371/journal.pone.0090737)

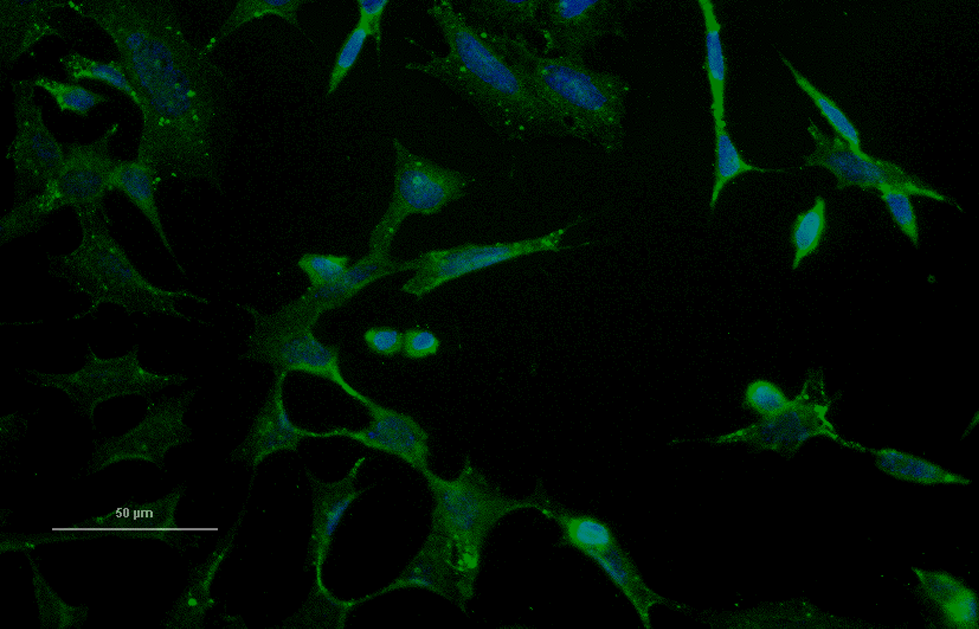

Supplement: Figure S1 — Expression of γ-synuclein in RGC5 revealed by indirect immunofluorescence RGC-5 cells were fixed, permeabilised, blocked and incubated with sheep polyclonal anti γ-synuclein abs. Subsequently the cells were incubated with rabbit anti sheep IgG-H&L conjugated with FITC. Nuclei staining were performed with DAPI and cells were visualized with a fluorescence microscope. γ-synuclein was expressed in all cells and it seems to be distributed in the cytoplasm. (TIF) [file pone.0090737.s001.tif]

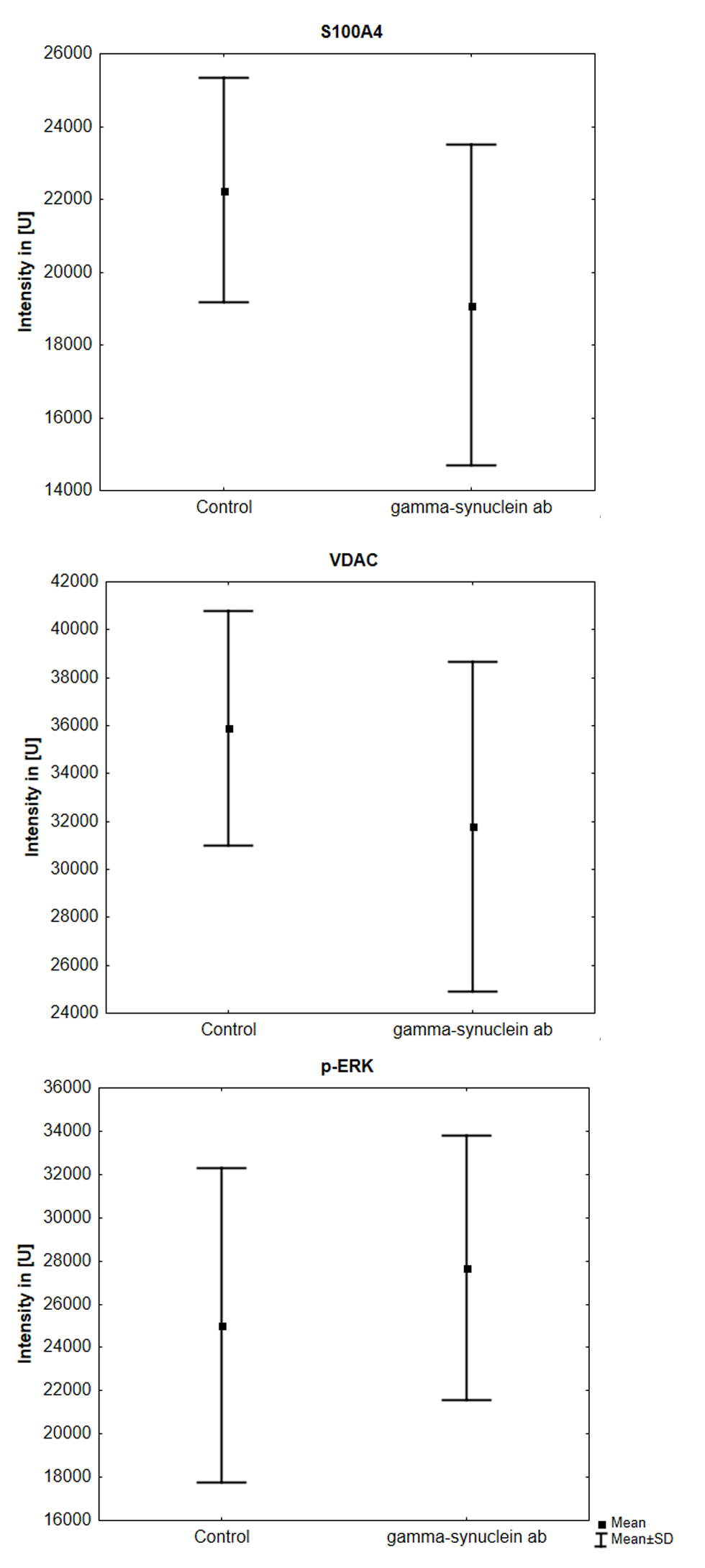

Supplement: Figure S2 — Regulation of mitochondrial apoptosis associated proteins. RGC-5 cells were preincubated with 0.5 µg/ml γ-synuclein abs and subsequently lyzed, tryptically digested before protein analysis via Microarray was performed. The differences were calculated in comparison to control cells, which were untreated. (n = 12, * = p<0.05; **p<0.01). (TIF) [file pone.0090737.s002.tif]
