# Supplementary material for: γ-Synuclein Antibodies Have Neuroprotective Potential on Neuroretinal Cells via Proteins of the Mitochondrial Apoptosis Pathway
Source: PLoS One. 2014 Mar 3;9(3):e90737. doi: 10.1371/journal.pone.0090737 (PMC3940944; doi:10.1371/journal.pone.0090737)
Supplement: Table S1 — Significant protein changes in γ-synuclein antibody treated RGC-5. (DOC) [file pone.0090737.s003.doc]

| **Swiss-Prot ID** | **Entrez Gene Name** | **Location** | **Type(s)** | **Fold Change** |
| --- | --- | --- | --- | --- |
| P09671 | superoxide dismutase 2, mitochondrial | Cytoplasm | enzyme | -190.056 |
| P02666 | casein beta | Extracellular Space | kinase | -67.789 |
| Q9CQX2 | cytochrome b5 type B (outer mitochondrial membrane) | Cytoplasm | enzyme | -14.927 |
| Q9QYR9 | acyl-CoA thioesterase 2 | Cytoplasm | enzyme | -8.921 |
| P62075 | translocase of inner mitochondrial membrane 13 homolog (yeast) | Cytoplasm | transporter | -8.649 |
| Q9R0P5 | destrin (actin depolymerizing factor) | Cytoplasm | other | -8.505 |
| Q924C1-1 | exportin 5 | Nucleus | transporter | -7.036 |
| P20152 | vimentin | Cytoplasm | other | -6.789 |
| Q99LS3 | phosphoserine phosphatase | Cytoplasm | phosphatase | -5.885 |
| Q9CR86 | calcium regulated heat stable protein 1, 24kDa | Cytoplasm | other | -5.517 |
| D3Z3G6 | mitogen-activated protein kinase 3 | Cytoplasm | kinase | -4.778 |
| P08074 | carbonyl reductase 2 | Cytoplasm | enzyme | -4.745 |
| Q62426 | cystatin B (stefin B) | Cytoplasm | other | -4.634 |
| Q3TW96-1 | UDP-N-acteylglucosamine pyrophosphorylase 1-like 1 | unknown | other | -4.618 |
| P31786 | diazepam binding inhibitor (GABA receptor modulator, acyl-CoA binding protein) | Cytoplasm | other | -4.572 |
| Q3TTN3 | voltage-dependent anion channel 3 | Cytoplasm | ion channel | -4.483 |
| Q8R1I1 | ubiquinol-cytochrome c reductase, complex III subunit X | Cytoplasm | enzyme | -4.38 |
| A2AEY2 | four and a half LIM domains 1 | Cytoplasm | other | -4.251 |
| P07091 | S100 calcium binding protein A4 | Cytoplasm | other | -4.193 |
| O35900 | LSM2 homolog, U6 small nuclear RNA associated (S. cerevisiae) | Nucleus | other | -4.184 |
| Q8BH95 | enoyl CoA hydratase, short chain, 1, mitochondrial | Cytoplasm | enzyme | -3.679 |
| P35279-1 | RAB6A, member RAS oncogene family | Cytoplasm | enzyme | -3.666 |
| P00375 | dihydrofolate reductase | unknown | enzyme | -3.552 |
| Q8C1B7-1 | septin 11 | Nucleus | other | -3.436 |
| A8C1V1 | mesencephalic astrocyte-derived neurotrophic factor | Extracellular Space | other | -3.414 |
| Q05920 | pyruvate carboxylase | Cytoplasm | enzyme | -3.383 |
| P08113 | heat shock protein 90kDa beta (Grp94), member 1 | Cytoplasm | other | -3.369 |
| Q91WQ3 | tyrosyl-tRNA synthetase | Cytoplasm | enzyme | -3.306 |
| P48678-1 | lamin A/C | Nucleus | other | -3.297 |
| Q8VCW8 | acyl-CoA synthetase family member 2 | Cytoplasm | enzyme | -3.284 |
| Q62465 | vesicle amine transport protein 1 homolog (T. californica) | Plasma Membrane | transporter | -3.216 |
| Q05816 | fatty acid binding protein 5 (psoriasis-associated) | Cytoplasm | transporter | -3.088 |
| P16110 | lectin, galactoside-binding, soluble, 3 | Extracellular Space | other | -3.053 |
| P70296 | phosphatidylethanolamine binding protein 1 | Cytoplasm | other | -3.027 |
| P41731 | CD63 molecule | Plasma Membrane | other | -3.02 |
| E9QLV8 | aldehyde dehydrogenase 1 family, member L2 | Cytoplasm | enzyme | -3.012 |
| P20029 | heat shock 70kDa protein 5 (glucose-regulated protein, 78kDa) | Cytoplasm | enzyme | -2.97 |
| Q9DBP5 | cytidine monophosphate (UMP-CMP) kinase 1, cytosolic | Nucleus | kinase | -2.959 |
| P70202 | latexin | Cytoplasm | other | -2.93 |
| Q920E5 | farnesyl diphosphate synthase | Cytoplasm | enzyme | -2.913 |
| P08003 | protein disulfide isomerase family A, member 4 | Cytoplasm | enzyme | -2.9 |
| E9Q9H9 | peroxiredoxin 5 | Cytoplasm | enzyme | -2.868 |
| P05201 | glutamic-oxaloacetic transaminase 1, soluble (aspartate aminotransferase 1) | Cytoplasm | enzyme | -2.857 |
| Q9D1D4-1 | transmembrane emp24-like trafficking protein 10 (yeast) | Cytoplasm | transporter | -2.847 |
| Q8VDN2 | ATPase, Na+/K+ transporting, alpha 1 polypeptide | Plasma Membrane | transporter | -2.837 |
| Q9CVB6 | actin related protein 2/3 complex, subunit 2, 34kDa | Cytoplasm | other | -2.796 |
| Q9D892 | inosine triphosphatase (nucleoside triphosphate pyrophosphatase) | Cytoplasm | enzyme | -2.765 |
| P45878 | FK506 binding protein 2, 13kDa | Cytoplasm | enzyme | -2.745 |
| O08807 | peroxiredoxin 4 | Cytoplasm | enzyme | -2.744 |
| Q9CQB5 | CDGSH iron sulfur domain 2 | Cytoplasm | other | -2.685 |
| P59999 | actin related protein 2/3 complex, subunit 4, 20kDa | unknown | other | -2.671 |
| Q9ERK4 | CSE1 chromosome segregation 1-like (yeast) | Nucleus | transporter | -2.671 |
| Q9CQB4 | ubiquinol-cytochrome c reductase binding protein | Cytoplasm | enzyme | -2.659 |
| Q61024 | asparagine synthetase (glutamine-hydrolyzing) | Cytoplasm | enzyme | -2.648 |
| Q60930 | voltage-dependent anion channel 2 | Cytoplasm | ion channel | -2.64 |
| P12382 | phosphofructokinase, liver | Cytoplasm | kinase | -2.631 |
| P56135 | ATP synthase, H+ transporting, mitochondrial Fo complex, subunit F2 | Cytoplasm | transporter | -2.58 |
| P16045 | lectin, galactoside-binding, soluble, 1 | Extracellular Space | other | -2.525 |
| P61021 | RAB5B, member RAS oncogene family | Cytoplasm | enzyme | -2.514 |
| Q9CR51 | ATPase, H+ transporting, lysosomal 13kDa, V1 subunit G1 | Cytoplasm | transporter | -2.501 |
| Q9D358-1 | acid phosphatase 1, soluble | Cytoplasm | phosphatase | -2.492 |
| Q3TN39 | solute carrier family 3 (activators of dibasic and neutral amino acid transport), member 2 | Plasma Membrane | transporter | -2.483 |
| P47754 | capping protein (actin filament) muscle Z-line, alpha 2 | Cytoplasm | other | -2.467 |
| P40142 | transketolase | Cytoplasm | enzyme | -2.459 |
| E0CXH5 | triosephosphate isomerase 1 | Cytoplasm | enzyme | -2.457 |
| Q9DCT8 | cysteine-rich protein 2 | Plasma Membrane | other | -2.448 |
| Q9DCN2-1 | cytochrome b5 reductase 3 | Cytoplasm | enzyme | -2.427 |
| Q8BH04 | phosphoenolpyruvate carboxykinase 2 (mitochondrial) | Cytoplasm | kinase | -2.418 |
| P68037 | ubiquitin-conjugating enzyme E2L 3 | Cytoplasm | enzyme | -2.389 |
| Q61425 | hydroxyacyl-CoA dehydrogenase | Cytoplasm | enzyme | -2.36 |
| D3Z3A8 | myosin IXA | Cytoplasm | enzyme | -2.36 |
| Q9JIF7 | coatomer protein complex, subunit beta 1 | Cytoplasm | transporter | -2.351 |
| Q60932-1 | voltage-dependent anion channel 1 | Cytoplasm | ion channel | -2.345 |
| Q07813 | BCL2-associated X protein | Cytoplasm | transporter | -2.337 |
| Q99PT1 | Rho GDP dissociation inhibitor (GDI) alpha | Cytoplasm | other | -2.328 |
| Q3UG45 | solute carrier family 7 (amino acid transporter light chain, L system), member 5 | Plasma Membrane | transporter | -2.282 |
| Q9CQI6 | coactosin-like 1 (Dictyostelium) | Cytoplasm | other | -2.279 |
| P17710-1 | hexokinase 1 | Cytoplasm | kinase | -2.276 |
| P62715 | protein phosphatase 2, catalytic subunit, beta isozyme | Cytoplasm | phosphatase | -2.26 |
| P61924 | coatomer protein complex, subunit zeta 1 | Cytoplasm | transporter | -2.259 |
| P62835 | RAP1A, member of RAS oncogene family | Cytoplasm | enzyme | -2.239 |
| Q920A5 | serine carboxypeptidase 1 | Cytoplasm | peptidase | -2.237 |
| Q3V2H3 | sorting nexin 12 | unknown | transporter | -2.217 |
| P14211 | calreticulin | Cytoplasm | transcription regulator | -2.178 |
| Q9CPT4 | chromosome 19 open reading frame 10 | Extracellular Space | cytokine | -2.17 |
| P62334 | proteasome (prosome, macropain) 26S subunit, ATPase, 6 | Nucleus | peptidase | -2.168 |
| O35215 | D-dopachrome tautomerase | Cytoplasm | enzyme | -2.118 |
| Q66JR8 | parathymosin | Nucleus | other | -2.105 |
| P11031 | SUB1 homolog (S. cerevisiae) | Nucleus | transcription regulator | -2.104 |
| Q3UHX2 | PDGFA associated protein 1 | Cytoplasm | other | -2.09 |
| D4AFX7 | DnaJ (Hsp40) homolog, subfamily C, member 13 | unknown | other | -2.058 |
| P13020-1 | gelsolin | Extracellular Space | other | -2.051 |
| Q8BP47 | asparaginyl-tRNA synthetase | Cytoplasm | enzyme | -2.039 |
| Q9QXT0 | canopy 2 homolog (zebrafish) | Plasma Membrane | other | -2.037 |
| P84078 | ADP-ribosylation factor 1 | Cytoplasm | enzyme | -2.028 |
| E9Q120 | lon peptidase 1, mitochondrial | Cytoplasm | peptidase | -2.024 |
| P26443 | glutamate dehydrogenase 1 | Cytoplasm | enzyme | -2.023 |
| O08553 | dihydropyrimidinase-like 2 | Cytoplasm | enzyme | -2.01 |
| P23198 | chromobox homolog 3 | Nucleus | transcription regulator | 2.013 |
| P26043 | radixin | Cytoplasm | other | 2.013 |
| P49312-2 | heterogeneous nuclear ribonucleoprotein A1 | Nucleus | other | 2.022 |
| O54734 | dolichyl-diphosphooligosaccharide--protein glycosyltransferase | Cytoplasm | enzyme | 2.024 |
| P14685 | proteasome (prosome, macropain) 26S subunit, non-ATPase, 3 | Cytoplasm | other | 2.029 |
| P07724 | albumin | Extracellular Space | transporter | 2.042 |
| Q62167 | DEAD (Asp-Glu-Ala-Asp) box polypeptide 3, X-linked | Nucleus | enzyme | 2.062 |
| P14069 | S100 calcium binding protein A6 | Cytoplasm | transporter | 2.063 |
| P14206 | ribosomal protein SA | Cytoplasm | translation regulator | 2.077 |
| Q8VDM4 | proteasome (prosome, macropain) 26S subunit, non-ATPase, 2 | Cytoplasm | other | 2.094 |
| P29391 | ferritin, light polypeptide | Cytoplasm | other | 2.1 |
| O70404 | vesicle-associated membrane protein 8 (endobrevin) | Plasma Membrane | other | 2.105 |
| Q9Z1N5 | DEAD (Asp-Glu-Ala-Asp) box polypeptide 39B | Nucleus | enzyme | 2.124 |
| P62317 | small nuclear ribonucleoprotein D2 polypeptide 16.5kDa | Nucleus | other | 2.127 |
| P62309 | small nuclear ribonucleoprotein polypeptide G | Nucleus | other | 2.141 |
| P32067 | Sjogren syndrome antigen B (autoantigen La) | Nucleus | enzyme | 2.148 |
| D3YVD9 | 40S ribosomal protein S28 pseudogene | unknown | other | 2.154 |
| P84099 | ribosomal protein L19 | Cytoplasm | other | 2.16 |
| O55135 | eukaryotic translation initiation factor 6 | Cytoplasm | translation regulator | 2.162 |
| E9PXE7 | ribosomal protein S11 | Cytoplasm | other | 2.162 |
| Q497E1 | ribosomal protein S23 | Cytoplasm | translation regulator | 2.162 |
| Q99MR6-1 | serrate RNA effector molecule homolog (Arabidopsis) | Nucleus | other | 2.201 |
| P47911 | ribosomal protein L6 | Cytoplasm | other | 2.224 |
| P70698 | CTP synthase | Nucleus | enzyme | 2.239 |
| P35527 | keratin 9 | Cytoplasm | other | 2.265 |
| Q9Z0N1 | eukaryotic translation initiation factor 2, subunit 3 gamma, 52kDa | Cytoplasm | translation regulator | 2.285 |
| P80313 | chaperonin containing TCP1, subunit 7 (eta) | Cytoplasm | other | 2.305 |
| Q80V08 | ribosomal protein L17 | Cytoplasm | other | 2.308 |
| P35980 | ribosomal protein L18 | Cytoplasm | other | 2.322 |
| Q8BG05-2 | heterogeneous nuclear ribonucleoprotein A3 | Nucleus | other | 2.327 |
| Q8C0C7 | phenylalanyl-tRNA synthetase, alpha subunit | Cytoplasm | enzyme | 2.34 |
| Q9D6R2-1 | isocitrate dehydrogenase 3 (NAD+) alpha | Cytoplasm | enzyme | 2.34 |
| Q9EPU0-1 | UPF1 regulator of nonsense transcripts homolog (yeast) | Nucleus | enzyme | 2.398 |
| Q61937 | nucleophosmin (nucleolar phosphoprotein B23, numatrin) | Nucleus | transcription regulator | 2.4 |
| P62918 | ribosomal protein L8 | Cytoplasm | other | 2.402 |
| O55234 | proteasome (prosome, macropain) subunit, beta type, 5 | Cytoplasm | peptidase | 2.423 |
| P62307 | small nuclear ribonucleoprotein polypeptide F | Nucleus | other | 2.433 |
| Q60715-1 | prolyl 4-hydroxylase, alpha polypeptide I | Cytoplasm | enzyme | 2.467 |
| E9QMB1 | nascent polypeptide-associated complex alpha polypeptide | Nucleus | transcription regulator | 2.472 |
| Q3UEB3-1 | poly-U binding splicing factor 60KDa | Nucleus | other | 2.487 |
| P46061 | chondroadherin-like | Nucleus | other | 2.491 |
| Q3UM23 | ribonuclease/angiogenin inhibitor 1 | Cytoplasm | other | 2.495 |
| A2AGT5-1 | cytoskeleton associated protein 5 | Nucleus | other | 2.516 |
| Q9Z2X1-1 | heterogeneous nuclear ribonucleoprotein F | Nucleus | other | 2.522 |
| Q8K003 | coiled-coil domain containing 72 | unknown | other | 2.592 |
| P59325 | eukaryotic translation initiation factor 5 | Cytoplasm | translation regulator | 2.606 |
| P63242 | eukaryotic translation initiation factor 5A | Cytoplasm | translation regulator | 2.621 |
| Q9WUK2-1 | eukaryotic translation initiation factor 4H | Cytoplasm | translation regulator | 2.65 |
| P63037 | DnaJ (Hsp40) homolog, subfamily A, member 1 | Nucleus | other | 2.684 |
| P62849-1 | ribosomal protein S24 | Cytoplasm | other | 2.701 |
| Q9CX86 | heterogeneous nuclear ribonucleoprotein A0 | Nucleus | other | 2.715 |
| O09131 | glutathione S-transferase omega 1 | Cytoplasm | enzyme | 2.725 |
| P24547 | IMP (inosine 5'-monophosphate) dehydrogenase 2 | Cytoplasm | enzyme | 2.782 |
| P62192 | proteasome (prosome, macropain) 26S subunit, ATPase, 1 | Nucleus | peptidase | 2.79 |
| A0PJ96 | microtubule-associated protein 1B | Cytoplasm | other | 2.795 |
| B9EHS6 | mannosidase, alpha, class 2A, member 1 | Cytoplasm | enzyme | 2.811 |
| P09055 | integrin, beta 1 (fibronectin receptor, beta polypeptide, antigen CD29 includes MDF2, MSK12) | Plasma Membrane | transmembrane receptor | 2.857 |
| Q3UB15 | ribosomal protein L3 | Cytoplasm | other | 2.88 |
| P46471 | proteasome (prosome, macropain) 26S subunit, ATPase, 2 | Nucleus | peptidase | 2.919 |
| E9PYU6 | baculoviral IAP repeat containing 6 | Cytoplasm | enzyme | 2.931 |
| Q9EQU5-1 | SET nuclear oncogene | Nucleus | phosphatase | 2.936 |
| P62911 | ribosomal protein L32 | Cytoplasm | other | 2.942 |
| Q8BNI6 | translocase of outer mitochondrial membrane 70 homolog A (S. cerevisiae) | Cytoplasm | transporter | 2.963 |
| Q9EPL8 | importin 7 | Nucleus | transporter | 2.992 |
| P43276 | histone cluster 1, H1b | Nucleus | other | 3.017 |
| P15864 | histone cluster 1, H1c | Nucleus | other | 3.045 |
| P97855 | GTPase activating protein (SH3 domain) binding protein 1 | Nucleus | enzyme | 3.145 |
| P46935 | neural precursor cell expressed, developmentally down-regulated 4 | Cytoplasm | enzyme | 3.309 |
| Q8VHY0-1 | chondroitin sulfate proteoglycan 4 | Plasma Membrane | other | 3.424 |
| Q99M08 | predicted gene 2036 | unknown | other | 3.441 |
| E9PW43 | Sec61 beta subunit pseudogene | unknown | other | 3.512 |
| P43274 | histone cluster 1, H1e | Nucleus | other | 3.554 |
| P47963 | ribosomal protein L13 pseudogene 12 | Nucleus | other | 3.563 |
| Q6P8I4 | PEST proteolytic signal containing nuclear protein | Nucleus | other | 3.586 |
| P43275 | histone cluster 1, H1a | Nucleus | other | 3.592 |
| P62196 | proteasome (prosome, macropain) 26S subunit, ATPase, 5 | Nucleus | transcription regulator | 3.629 |
| E9PX78 | eukaryotic translation initiation factor 3, subunit B | Cytoplasm | translation regulator | 3.659 |
| A2ALM8 | B-cell receptor-associated protein 31 | Cytoplasm | transporter | 3.77 |
| Q9D7S7-1 | ribosomal protein L22-like 1 | unknown | other | 3.774 |
| Q8BH64 | EH-domain containing 2 | Nucleus | other | 3.785 |
| E9PWL3 | NHP2-like protein 1-like | unknown | other | 3.928 |
| Q61699-1 | heat shock 105kDa/110kDa protein 1 | Cytoplasm | other | 3.988 |
| Q9DAW9 | calponin 3, acidic | Cytoplasm | other | 4.14 |
| E9PW66 | nucleosome assembly protein 1-like 1 | Nucleus | other | 4.483 |
| P63276 | ribosomal protein S17 | Cytoplasm | other | 4.485 |
| P97379-1 | GTPase activating protein (SH3 domain) binding protein 2 | Nucleus | enzyme | 4.667 |
| Q62351 | transferrin receptor (p90, CD71) | Plasma Membrane | transporter | 4.678 |
| Q9JKB3-1 | cold shock domain protein A | Nucleus | transcription regulator | 4.708 |
| P00405 | cytochrome c oxidase subunit II | Cytoplasm | enzyme | 4.719 |
| Q8R1F1 | family with sequence similarity 129, member B | unknown | other | 4.727 |
| Q9CY58-1 | SERPINE1 mRNA binding protein 1 | Nucleus | other | 5.584 |
| P49817-1 | caveolin 1, caveolae protein, 22kDa | Plasma Membrane | other | 5.702 |
| P52293 | karyopherin alpha 2 (RAG cohort 1, importin alpha 1) | Nucleus | transporter | 5.741 |
| P34955 | serpin peptidase inhibitor, clade A (alpha-1 antiproteinase, antitrypsin), member 1 | Extracellular Space | other | 6.688 |
| P02769 | albumin | Extracellular Space | transporter | 8.714 |
| P97371 | proteasome (prosome, macropain) activator subunit 1 (PA28 alpha) | Cytoplasm | other | 9.865 |
| Q7TPV4 | MYB binding protein (P160) 1a | Nucleus | transcription regulator | 11.061 |
| Q9JIK5 | DEAD (Asp-Glu-Ala-Asp) box polypeptide 21 | Nucleus | enzyme | 12.577 |
| P12763 | alpha-2-HS-glycoprotein | Extracellular Space | other | 25.132 |
